# The New Institution for Epileptics in Berlin

**Published:** 1894-10-20

**Authors:** 


### The Institutional Workshop.

### HOSPITAL CONSTRUCTION.

### THE NEW INSTITUTION FOR EPILEPTICS IN BERLIN.

Berlin has, it would seem, taken the lead in the matter of providing the best accommodation for epileptics, by erecting a large establishment solely devoted to this class of patients. The whole thing has been done on a very large scale, and both the plan as a whole and the details are excellent. The new institution is situated about an hour's railway journey from the capital, and a special line of rails has been taken straight to the stores department. The home possesses a farm of some 250 acres, and the water supply is provided from the new Berlin waterworks, through the medium of separate waterworks, necessitated by the high position of the institution. There is accommodation for one thousand patients, and the institution is divided into three sections separated from each other. Furthest to the south is the school section, intended for a hundred children. It consists of three extensive two-storied buildings, and a fourth one-storied building, which forms an excellent hall for gymnastics. The roomy class-rooms are only used for the school teaching proper, there being separate apartments both for the girls' domestic work and for the boys' manual occupation In addition to these, there are capital eating, sitting and bed rooms, a residence for the inspector, rooms for governesses, attendants, &c. This department is in the charge of an "education inspector," with a fair amount of independence; a doctor being director for the whole of the institution.

A few hundred yards to the north, and occupying a central position as regards the whole set of buildings, is the section for the insane epileptics: there are two buildings, one for the male and one for the female patients, one on each side of the administration buildings, in which a number of joint apartments are situated, such as billiard-rooms, libraries, conversation and ball-rooms, &c., in connection with which is a pretty little theatre, with very complete appointments, and capable of seating 600 people. Considering that the institution is a municipal one, principally arranged for poor patients from Berlin, it must be acknowledged that everything has been done to brighten their unhappy existence. It is certainly anticipated that this excellent installation will also be patronised by paying patients, and for such an annual charge of 700 marks, or £45, will be made. It should, however, be noted that there will be only one class of board, and that the municipal and the private patients will be treated alike. The two buildings for more or less violent epileptics are arranged and fitted according to hospital requirements; in the closets, in the bath-rooms, and more especially in the cells, everything has been done to insure against the patients doing any harm to themselves or their clothes, and to protect the rooms and the furniture from being destroyed. In order to effect this numerous alterations have been necessary during the process of building as regards windows, doors, locks, &c., but the ultimate result appears to be exceedingly satisfactory, The cell has an ordinary outer window, whereas the inner window is of wrought iron, paned with unbreakable glass; the hinges are so arranged that the surface towards the room is perfectly smooth. Between the two windows is a curtain, which can be drawn from the outside when the patients want rest. The electric lamp is placed in the wall behind a very thick pane of glass, and from the corridors the light can be regulated to six different degrees of strength. There is no panelling, &c., but only cemented aud painted walls. In the parlours or assembly rooms there is made no attempt to protect the windows from being broken from the inside, but outside there are ornamental wrought iron railings, which also afford room at the bottom for flowering plants. Plants are altogether very extensively used in Berlin's asylums and hospitals.

Furthest north is the most pleasing part of the large institution, quite a little village, consisting of sixteen two-storied villas, every house appointed as an ordinary residence, capable of accommodating twenty-five to fifty patients. These are all slighter cases, where the epilepsy has not much affected the mental and bodily faculties. They are, therefore, all intended to be kept in employment, also for curative reasons, as far as possible in the open air, in the fields and garden whenever the weather will permit. In connection with this section is a large conservatory and a somewhat smaller hothouse, which are intended to supply the plants for the numerous windows of the institution. There is also a covered skittle-alley for the amusement of the male patients. To the west of the village lies the home farm, with accommodation for 6 horses, 64 cows, and about 150 pigs, There are also houses, for fowls, ducks, and pigeons. One of the "female" villas is located quite close to the farm, because it is more especially the women who are intended to work there.

To the west of the central section are the laundry, engine house, water tower, &c. There are ten boilers of thirty feet length, sixteen tinned copper vessels of 300 litre capacity each for the cooking of the food, several kitcheners, warming apparatuses, &c. The laundry, with its ingenious drying arrangements, is deserving of mention. The bath-house is also remarkably well appointed; the most striking feature is the swimming bath, 30 feet long, 20 feet broad, and five feet deep, lined with porcelain tiles; the water can be heated or cooled to any temperature. In connection with the institution is a small infirmary, and the handsome and roomy church is provided with several "fit rooms" for patients taken ill during service. Electric lighting is installed everywhere. The school section is heated with warm water, the other sections by a combined steam and hot-air system. The work is excellent throughout the building and the control has been exceptionally keen.

The whole cost of the institution is about £300,000, or some £300 for each patient accommodated.

